# A20 protects cells from TNF-induced apoptosis through linear ubiquitin-dependent and -independent mechanisms

**DOI:** 10.1038/s41419-019-1937-y

**Published:** 2019-09-18

**Authors:** Dario Priem, Michael Devos, Sarah Druwé, Arne Martens, Karolina Slowicka, Adrian T. Ting, Manolis Pasparakis, Wim Declercq, Peter Vandenabeele, Geert van Loo, Mathieu J. M. Bertrand

**Affiliations:** 1 Center for Inflammation Research, VIB, Ghent, Belgium; 2 Department of Biomedical Molecular Biology, Ghent University, Ghent, Belgium; 3 Precision Immunology Institute, Icahn School of Medicine at Mount Sinai, New York, NY USA; 4 Institute for Genetics, Cologne Excellence Cluster on Cellular Stress Responses in Aging-Associated Diseases (CECAD) and Center for Molecular Medicine, University of Cologne, Cologne, Germany

**Keywords:** Ubiquitins, Apoptosis, Stress signalling

## Abstract

The cytokine TNF promotes inflammation either directly by activating the MAPK and NF-κB signaling pathways, or indirectly by triggering cell death. A20 is a potent anti-inflammatory molecule, and mutations in the gene encoding A20 are associated with a wide panel of inflammatory pathologies, both in human and in the mouse. Binding of TNF to TNFR1 triggers the NF-κB-dependent expression of A20 as part of a negative feedback mechanism preventing sustained NF-κB activation. Apart from acting as an NF-κB inhibitor, A20 is also well-known for its ability to counteract the cytotoxic potential of TNF. However, the mechanism by which A20 mediates this function and the exact cell death modality that it represses have remained incompletely understood. In the present study, we provide in vitro and in vivo evidences that deletion of A20 induces RIPK1 kinase-dependent and -independent apoptosis upon single TNF stimulation. We show that constitutively expressed A20 is recruited to TNFR1 signaling complex (Complex I) via its seventh zinc finger (ZF7) domain, in a cIAP1/2-dependent manner, within minutes after TNF sensing. We demonstrate that Complex I-recruited A20 protects cells from apoptosis by stabilizing the linear (M1) ubiquitin network associated to Complex I, a process independent of its E3 ubiquitin ligase and deubiquitylase (DUB) activities and which is counteracted by the DUB CYLD, both in vitro and in vivo. In absence of linear ubiquitylation, A20 is still recruited to Complex I via its ZF4 and ZF7 domains, but this time protects the cells from death by deploying its DUB activity. Together, our results therefore demonstrate two distinct molecular mechanisms by which constitutively expressed A20 protect cells from TNF-induced apoptosis.

## Introduction

TNF is a well-established inducer of inflammation, and a pharmacological target in several inflammatory disorders^1^. Binding of TNF to TNFR1 induces the rapid assembly of a membrane-bound signaling complex known as TNFR1 Complex I. The initial binding of TRADD and RIPK1 to the receptor allows the subsequent recruitment of TRAF2 and of the E3 ubiquitin ligases cIAP1/2 and LUBAC. Together, these E3s generate a dense network of ubiquitin chains resulting in the stabilization of Complex I and in the recruitment of the kinases that activate the MAPKs and NF-κB signaling pathways^2,3^. The K63-ubiquitin chains generated by cIAP1/2 operate as binding stations for the adapter proteins TAB2/3 and for the recruitment of the kinase TAK1, which subsequently activates the MAPK pathway by phosphorylation^3–5^. On the other hand, the multiprotein E3 complex LUBAC (composed of Sharpin, HOIP, and HOIL-1) docks on these newly formed K63-linked ubiquitin chains and further conjugates Complex I components with linear(M1)-ubiquitin chains; in this way creating, in some cases, hybrid K63/M1 chains^2,6^. The adapter protein NEMO binds to these M1-ubiquitin chains and brings the kinases IKKα and IKKβ to the complex. The close proximity between TAK1 and IKKα/β on the hybrid K63/M1 chains permits activation of IKKα/β by TAK1, and the subsequent IKKα/β-dependent activation of the NF-κB pathway^7^. NEMO also enables the recruitment of the noncanonical kinases TBK1/IKKε to the complex, which may serve to counteract IKKα/β-mediated NF-κB activation^8–10^. Together, the MAPK and NF-κB pathways drive the transcription of a large set of genes, including some encoding prosurvival or proinflammatory molecules. The ubiquitin network associated to Complex I is negatively regulated by a subset of deubiquitylases (DUBs), including A20 and CYLD, whose function consists in destabilizing the signaling complex and restricting signaling to MAPKs and NF-κB^11^.

The pathologic role of TNF in inflammatory disorders has long been attributed to the MAPK/NF-κB-dependent induction of proinflammatory mediators, but recent studies in mice have demonstrated that TNF also indirectly causes inflammation by inducing cell death, in the form of apoptosis and necroptosis^12^. Cell death is however not the default response of most cells to TNF, and is only induced when protective brakes in the pathway are inactivated^12,13^. The NF-κB-dependent induction of prosurvival molecules serves as a late brake protecting cells against RIPK1 kinase-independent apoptosis, while the IKKα/β- and TBK1/IKKε-dependent phosphorylation of RIPK1 functions as independent early brakes preventing RIPK1 kinase-dependent apoptosis^9,10,14^. Consequently, inactivation of any of these brakes suffices to switch the TNF response from survival to caspase-8-mediated apoptosis. This involves dissociation of TRADD or RIPK1 from Complex I and their subsequent association with FADD and caspase-8 to form the cytosolic Complex IIa or IIb, respectively^15^. Necroptosis occurs when caspase-8 activation fails or is inhibited, and involves RIPK1 kinase-dependent recruitment of RIPK3 and MLKL to Complex II to form the necrosome.

Mutations in the gene encoding A20 are associated with a wide panel of inflammatory pathologies, both in human and in the mouse^16,17^. Apart from functioning as an inhibitor of the NF-κB pathway, A20 is also known to potently inhibit TNF cytotoxicity^18–20^. This suggests that the inflammatory disorders associated with mutations in A20 may be caused, at least in part, by excessive cell death induction. Our understanding of the anti-death role of A20 has however remained limited. Structurally, A20 consists out of a N-terminal ovarian tumor (OTU) domain, conferring it K48- and K63-DUB activity^21–23^, and seven conserved C-terminal zinc finger (ZF) domains. The ZF region allows recruitment of A20 to ubiquitin scaffolds in immune signaling complexes, with ZF4 having highest affinity for K63 chains, and ZF7 preferentially binding to M1 chains^24–29^. In addition, the ZF4 is also reported to provide E3 ubiquitin ligase activity^27,30–32^. So far, A20 was proposed to protect cells from apoptosis by deubiquitylating caspase-8, and necroptosis by deubiquitylating RIPK3^33,34^. However, an inactivating mutation in the OTU domain of A20 does not suffice to switch the TNF response from survival to apoptosis^24,27,35^. This is in line with the fact that mice bearing the same A20 mutation do not present any cell death phenotype^25,36^. Other studies have instead proposed the prosurvival function of A20 to be mediated by its ZF7^24,35,37^, by binding and protecting Complex I-associated M1-ubiquitin chains from degradation^24^. It, however, remains unclear whether a mutated ZF7 suffices to switch the TNF response to death since the authors only reported a significant effect of the mutation in conditions affecting additional prosurvival brakes in the pathway, such as caspase- or protein translation-inhibited conditions.

In this study, we further characterized the anti-death role of A20 in the TNF pathway. We found that A20-deficiency results in RIPK1 kinase-dependent and -independent apoptosis upon single TNF stimulation, and demonstrate two distinct mechanisms by which constitutively expressed A20 protects the cells: one relying on A20′s ability to stabilize M1-ubiquitin chains in Complex I, and another one, revealed in absence of M1-ubiquitin chains, which requires A20′s DUB activity.

## Results

### A20-deficiency triggers RIPK1 kinase-dependent and -independent apoptosis upon single TNF stimulation

**Fig. 1:**
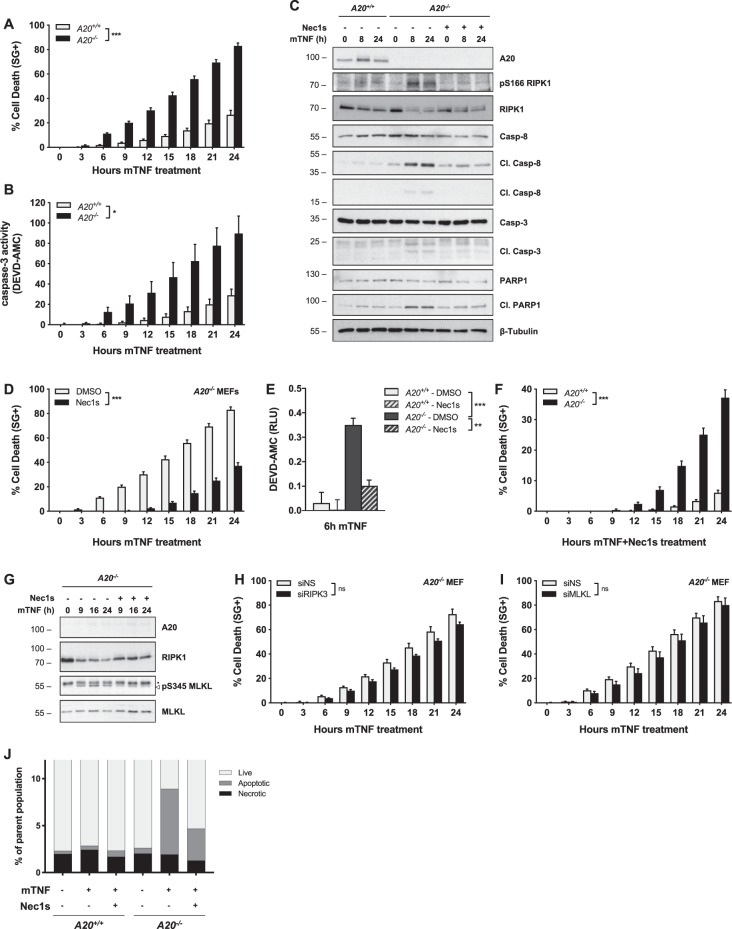
A20 deficiency in MEFs triggers RIPK1 kinase-dependent and -independent apoptosis, but not necroptosis, following single TNF stimulation. **a**–**g** *A20*^*+/+*^ and *A20*^−*/*−^ MEFs were pretreated with the indicated compounds for 30 min before stimulation with 10 ng/ml mTNF. Cell death was measured in function of time by SytoxGreen (SG) positivity (**a**, **d**, **f**) and cell death markers were monitored by immunoblotting (**c**, **g**). Extracellular caspase activity was quantified using a fluorescent caspase-activity probe (DEVD-AMC) (**b**). Intracellular caspase activity was quantified using a fluorescent caspase-activity probe (DEVD-AMC) after lysing the cells (**e**). **h**, **i** *A20*^*−/−*^ MEFs were transfected with siRNA targeting RIPK3 (H) or MLKL (**i**) or nonspecific siRNA (NS). Cells were pretreated with the indicated compounds for 30 min before stimulation with 10 ng/ml mTNF. Cell death was measured in function of time by SytoxGreen (SG) positivity. **j** *A20*^*+/+*^ and *A20*^−*/*−^ MEFs were pretreated with the indicated compounds for 30 min before stimulation with 10 ng/ml mTNF. The occurrence of apoptotic or necroptotic cell death was determined by FACS analysis. FACS data represent a representative experiment from three independent experiments. Cell death experiments are presented as mean ± SEM of three independent experiments. Statistical significance for the cell death assays was determined using two-way ANOVA followed by a Tukey post hoc test. Significance between samples is indicated in the figure as follows: *p < 0.05; **p < 0.01; ***p < 0.001; ns non significant.

Although A20-deficiency promotes NF-κB activation and thereby enforces a protective brake in the TNFR1 pathway, loss of A20 paradoxically sensitizes cells to TNF-induced death. To get a better understanding of A20′s anti-death role, we evaluated the cell death modality induced by A20 deletion in MEFs. A20-deficiency was sufficient to greatly sensitize the cells to death upon single TNF stimulation (Fig. 1a). The cell death was characterized by the release of active caspase-3 (Fig. 1b) and the presence of cleaved caspase-8, caspase-3, and PARP-1 in the cell lysates (Fig. 1c), indicative of apoptosis. Of note, A20-deficiency did not sensitize MEFs to apoptosis induced by staurosporin, etoposide, or tunicamycin (Fig. S1A). Interestingly, we detected RIPK1 activation (monitored by S166 autophosphorylation) in the lysate of TNF-stimulated A20-deficient MEFs (Fig. 1c), suggestive of RIPK1 kinase-dependent cell death. Inhibition of RIPK1 by Necrostatin-1s (Nec1s) resulted in reduced, but not totally inhibited, caspase activation and cell death, demonstrating simultaneous occurrence of RIPK1 kinase-dependent and -independent apoptosis (Fig. 1c–f).

Although hallmarks of apoptosis were primarily detected, we also observed RIPK1 kinase-dependent phosphorylation of MLKL on S345 (Fig. 1g), a reported marker of necroptosis^38^. To address if necroptosis was simultaneously occurring in these cells, we blocked the necroptotic pathway by knocking down RIPK3 and MLKL. While repressing RIPK3 and MLKL effectively repressed necroptosis induced by TNF in presence of the pan-caspase inhibitor zVAD-fmk (zVAD) (Fig. S1B, C), it did not significantly influence the extent of cell death induced by single TNF stimulation in *A20*^−/−^ MEFs (Fig. 1h, i). Single-cell analysis combining a general cell death probe with a caspase-activity probe, also excluded the presence of purely necrotic cells in *A20*^*−/−*^ MEFs stimulated with TNF (Fig. 1j, Fig. S1D, E). Together, our results demonstrate that, despite activation of a necroptotic marker, A20-defiency in MEFs triggers RIPK1 kinase-dependent and -independent apoptosis upon single TNF stimulation.

### A20 provides in vitro and in vivo protection to intestinal epithelial cells against TNF-induced RIPK1 kinase-dependent and -independent apoptosis

**Fig. 2:**
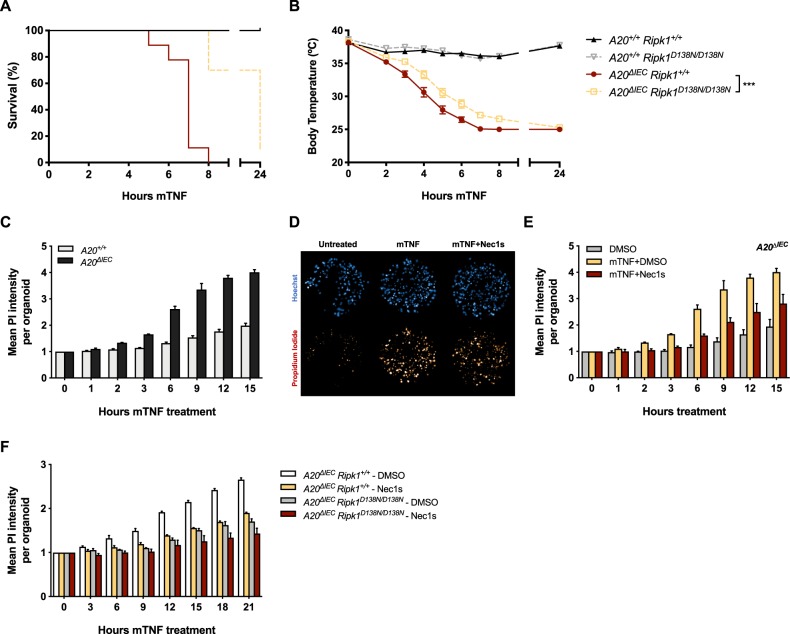
A20 protects intestinal epithelial cells in vitro and in vivo against TNF-induced RIPK1 kinase-dependent and -independent apoptosis. **a**, **b** *A20*^*+/+*^ *Ripk1*^*+/+*^ (*n* = 7), *A20*^*+/+*^ *Ripk1*^*D138N/D138N*^ (*n* = 5), *A20*^*ΔIEC*^ *Ripk1*^*+/+*^ (*n* = 9), and *A20*^*ΔIEC*^ *Ripk1*^*D138N/D138N*^ (*n* = 9) were injected i.p. with 5 µg/20 g mTNF. Cumulative survival rates (**a**) and body temperature (**b**) were determined in function of time. Temperatures are presented as mean ± SEM. Statistical significance for body temperatures of the mice was determined using two-way ANOVA followed by a Tukey post hoc test. Survival curves were compared using log-rank Mantel–Cox test. **c**–**e** Primary intestinal organoid cultures were obtained from *A20*^*+/+*^ and *A20*^*ΔIEC*^ mice and pretreated with the indicated compounds for 30 min before stimulation with 10 ng/ml mTNF. Cell death was measured by propidium iodide (PI) and is plotted as the relative mean PI intensity per organoid. Data represent a representative experiment from three independent experiments and are presented as mean ± SD. **d** Representative images for *A20*^*ΔIEC*^ organoid cultures stained with Hoechst and PI after 6 h of mTNF stimulation. **f** Primary intestinal organoid cultures were obtained from mice with indicated genotypes and pretreated with the indicated compounds for 30 min before stimulation with 10 ng/ml mTNF. Cell death was measured by propidium iodide (PI) and is plotted as the relative mean PI intensity per organoid. Data represent a representative experiment from three independent experiments and are presented as mean ± SD. Significance between samples is indicated in the figure as follows: *p < 0.05; **p < 0.01; ***p < 0.001; ns non significant.

To evaluate whether the results obtained in MEFs could be extrapolated to other cell types and to an in vivo context, we made use of mice specifically lacking A20 in intestinal epithelial cells (IECs) (*A20*^*ΔIEC*^)^39^. These mice die shortly after administrating low doses of TNF due to hyperinflammation and/or extensive TNF-dependent death of IECs^39^. We found that crossing these mice with mice expressing kinase-dead RIPK1 (*Ripk1*^*D138N/D138N*^)^40^ provided partial protection to TNF-induced lethality. Indeed, *A20*^*ΔIEC*^ *Ripk1*^*D138N/D138N*^ mice showed significant delay in body temperature drop and associated lethality when compared to the *A20*^*ΔIEC*^ *Ripk1*^*+/+*^ littermates (Fig. 2a, b). This partial protection was not resulting from inhibition of necroptosis since crossing the *A20*^*ΔIEC*^ mice with the *Ripk3*^*−/−*^ mice^41^ did not provide any protection (Fig. S2A). We also found that organoid cultures isolated from *A20*^*ΔIEC*^ mice died upon single TNF stimulation (Fig. 2c), and that the cell death could partially be prevented by pharmacological or genetic inhibition of RIPK1 kinase activity (Fig. 2d–f). Taken together, these data demonstrate a critical role for A20 in the in vitro and in vivo protection of intestinal epithelial cells against TNF-induced RIPK1 kinase-dependent and -independent apoptosis.

### Constitutively expressed A20 exerts its antideath function through cIAP1/2-dependent recruitment to TNFR1 Complex I

**Fig. 3:**
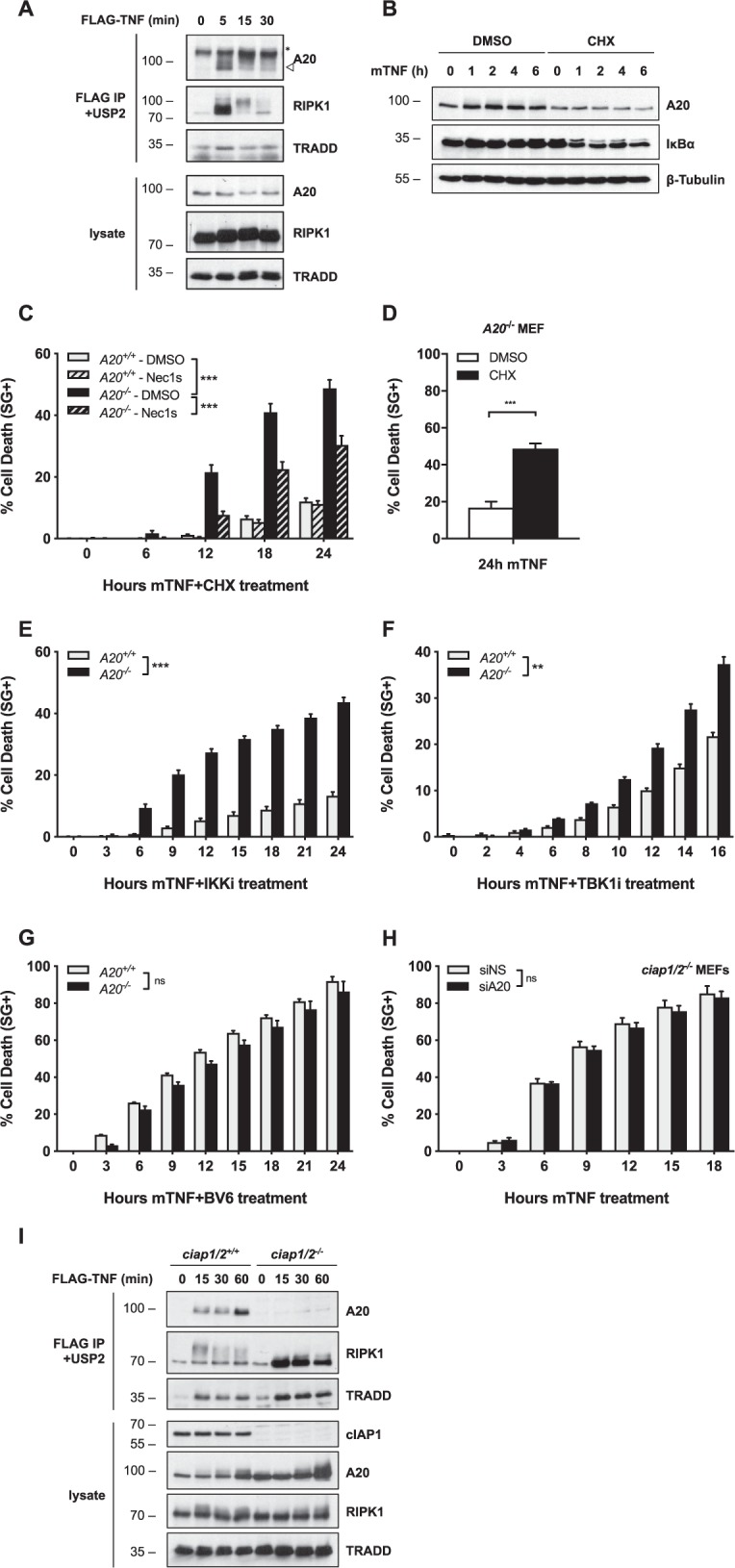
Constitutively expressed A20 exerts its antideath function through cIAP1/2-dependent recruitment to TNFR1 Complex I. **a** *A20*^*+/+*^ MEFs were stimulated with 1 µg/ml FLAG-hTNF for the indicated duration. TNFR1 Complex I was FLAG-immunoprecipitated, followed by USP2 treatment on the post-IPs. Protein levels were determined by immunoblotting. **b** *A20*^*+/+*^ MEFs were pretreated with CHX for 30 min before stimulation with 10 ng/ml mTNF for the indicated duration. Protein levels were determined by immunoblotting. **c**–**g** *A20*^*+/+*^ and *A20*^*−/−*^ MEFs were pretreated with the indicated compounds for 30 min before stimulation with 100 pg/ml (**c**–**f**) or 1 ng/ml (**g**) mTNF. Cell death was measured in function of time by SytoxGreen (SG) positivity. **h** *ciap1/2*^*−/−*^ MEFs were transfected with siRNA targeting A20 or nonspecific siRNA (NS). Cells were stimulated with 1 ng/ml mTNF. Cell death was measured in function of time by SytoxGreen (SG) positivity. **i** *ciap1/2*^*+/+*^ and *ciap1/2*^*−/−*^ MEFs were stimulated with 1 µg/ml FLAG-hTNF for the indicated duration. TNFR1 Complex I was FLAG-immunoprecipitated, followed by USP2 treatment on the post-IPs. Protein levels were determined by immunoblotting. Cell death experiments are presented as mean ± SEM of three independent experiments. Statistical significance for the cell death assays was determined using two-way ANOVA followed by a Tukey post hoc test. Significance between samples is indicated in the figure as follows: *p < 0.05; **p < 0.01; ***p < 0.001; ns non significant.

A20 is an NF-κB target gene and its inhibitory role on TNF-induced NF-κB is therefore widely seen as a negative-feedback mechanism preventing sustained NF-κB activation^42^. We found that constitutively expressed A20 is recruited to TNFR1 Complex I within minutes of TNF sensing (Fig. 3a), which is in line with previous reports^24,37^. To evaluate whether the antideath role of A20 originates from the constitutively expressed pool of A20 or from the one transcriptionally upregulated, we compared the cell death response of *A20*^*+/+*^ and *A20*^*−/−*^ MEFs to TNF in the presence of the translational inhibitor cycloheximide (CHX). The use of CHX indeed prevents the NF-κB-dependent induction of A20 in control MEFs (Fig. 3b), thereby allowing specific evaluation of the antideath role of the constitutively expressed A20. Remarkably, A20-deficiency still sensitized MEFs and HaCaT cells to RIPK1 kinase-dependent and -independent apoptosis following TNF + CHX treatment (Fig. 3c, Fig. S3A), which demonstrated the anti-death role of the constitutively expressed pool of A20 that is quickly recruited to Complex I. In addition, the cell death caused by A20 deficiency was not originating from sustained NF-κB activation, since inhibiting the NF-κB-dependent response by CHX did not protect but instead sensitize *A20*^*−/*^^*−*^ cells to TNF-induced death (Fig. 3d).

To position the antideath function of A20 in Complex I, we next evaluated the effect of A20 deletion in cells inactivated for other crucial antideath Complex I components, such as IKKα/β, TBK1, or the upstream regulators TAK1 and cIAP1/2^9,10,14,43^. We found that while A20-deficiency further sensitized cells in which IKKα/β, TBK1, or TAK1 had been inactivated (alone or together) (Fig. 3e, f, Fig. S3B, C), it did not increase the extent of death in cells where cIAP1/2 had been inactivated either pharmacologically or genetically (Fig. 3g, h). These results therefore indicate that A20 exerts its antideath function downstream of cIAP1/2, and upstream or parallel to the prosurvival roles played by IKKα/β, TBK1, and TAK1. In line with the notion that A20 relies on cIAP1/2 to mediate its prosurvival function, we observed that A20 was not recruited to TNFR1 Complex I in cIAP1/2-depleted conditions (Fig. 3i). Altogether, these results show that constitutively expressed A20 exerts its antiapoptotic function independently of NF-κB regulation, but through cIAP1/2-dependent recruitment to TNFR1 Complex I.

### Constitutively expressed A20 protects cells from TNF-induced death by stabilizing M1-ubiquitin chains in Complex I

**Fig. 4:**
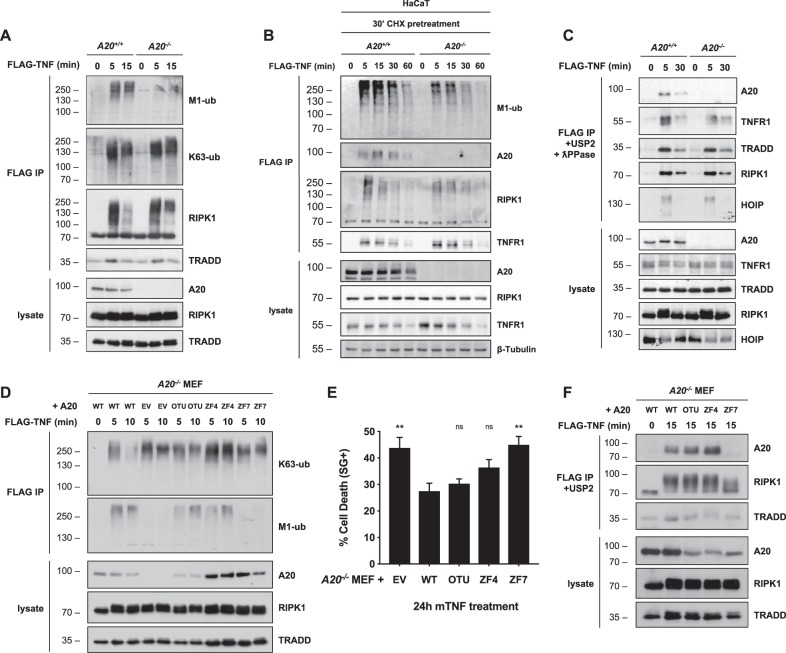
Constitutively expressed A20 protects cells from TNF-induced death via its ZF7 domain by stabilizing M1-linked ubiquitin chains in TNFR1 Complex I. **a** *A20*^*+/+*^ and *A20*^*−/−*^ MEFs were stimulated with 1 µg/ml FLAG-hTNF for the indicated duration. TNFR1 Complex I was subsequently FLAG-immunoprecipitated. Protein levels were determined by immunoblotting. **b** *A20*^*+/+*^ and *A20*^*−/−*^ HaCaTs were pretreated with CHX for 30 min before stimulation with 1 µg/ml FLAG-hTNF for the indicated duration. TNFR1 Complex I was subsequently FLAG-immunoprecipitated. Protein levels were determined by immunoblotting. **c** *A20*^*+/+*^ and *A20*^*−*^^*/−*^ MEFs were stimulated with 1 µg/ml FLAG-hTNF for the indicated duration. TNFR1 Complex I was FLAG-immunoprecipitated, followed by USP2 + ƛ-phosphatase treatment on the post-IPs. Protein levels were determined by immunoblotting. **d** *A20*^*−/−*^ MEFs lentivirally reconstituted with wild-type A20, empty vector (EV) or indicated A20 mutants were stimulated with 1 µg/ml FLAG-hTNF for the indicated duration. TNFR1 Complex I was subsequently FLAG-immunoprecipitated. Protein levels were determined by immunoblotting. **e** *A20*^*−/−*^ MEFs lentivirally reconstituted with wild-type A20, empty vector (EV) or indicated A20 mutants were stimulated with 1 ng/ml mTNF and cell death was measured in function of time by SytoxGreen (SG) positivity. **f** *A20*^*−/−*^ MEFs lentivirally reconstituted with wild-type A20, empty vector (EV) or indicated A20 mutants were stimulated with 1 µg/ml FLAG-hTNF for the indicated duration. TNFR1 Complex I was subsequently FLAG-immunoprecipitated, followed by USP2 treatment on the post-IPs. Protein levels were determined by immunoblotting. Cell death experiments are presented as mean ± SEM of three independent experiments. Statistical significance for the cell death assays was determined using one-way ANOVA followed by a Dunnett post hoc test. Significance between samples is indicated in the figure as follows: *p < 0.05; **p < 0.01; ***p < 0.001; ns non significant.

cIAP1/2 are at the origin of the ubiquitin network associated with Complex I. They generate K63-ubiquitin chains on TNFR1-associated adapter proteins that in turn allow the conjugation of M1 chains to Complex I components by LUBAC. Together, these ubiquitin chains ensure cell survival by recruiting TAK1, IKKα/β, and TBK1 to the TNFR1 signaling complex^7,9,10,14,43^. Having established that A20 exerts its antideath function downstream of cIAP1/2, we evaluated the effect of A20-deficiency on the ubiquitin network associated with Complex I. We found that *A20*^*−/−*^ MEFs have a clear defect in M1-ubiquitylation, as monitored by immunoblotting FLAG-immunoprecipitated Complex I for M1-ubiquitin or by direct immunoprecipitation of M1-ubiquitin chains using UBANs^7^ (Fig. 4a, S4A). The difference in M1-ubiquitylation between A20 proficient and deficient cells was maintained even upon CHX pretreatment, demonstrating that constitutively expressed A20 is responsible to maintain high levels of linear ubiquitin chains in Complex I (Fig. 4b). In contrast, A20-deficiency led to more K63-ubiquitylated proteins in Complex I, in part illustrated by RIPK1's ubiquitylation pattern (Fig. 4a). Treatment of immunoprecipitated Complex I with the deubiquitylase USP2 and with the λ-phosphatase demonstrated that the defects in ubiquitylation were not the result of impaired recruitment of the LUBAC subunit HOIP, or of the ubiquitylated substrates RIPK1, TRADD, or TNFR1 (Fig. 4c). To evaluate the domains of A20 implicated in the observed phenotype, we stably reconstituted A20-deficient MEFs with wild type (WT) A20 or with versions of A20 mutated in the OTU (DUB inactive), ZF4 (E3 inactive), or ZF7. We found that mutating any of these domains led to an accumulation of K63-ubiquitin chains in Complex I, but that only the ZF7 mutant additionally diminished M1-ubiquitylation (Fig. 4d), thereby further mimicking full loss of A20. In order to correlate the defects in ubiquitylation to cell death induction, we monitored the death response of these cells to single TNF stimulation. Interestingly, the ZF7 mutant was the only one recapitulating the sensitization observed in cells depleted of A20 (Fig. 4e). Indeed, the cell death did not significantly differ between cells re-expressing WT A20 and the ZF4- or OTU-mutants (Fig. 4e). We also found that mutating the ZF7 domain, but not the ZF4 or OTU domains, prevented recruitment of A20 to TNFR1 Complex I (Fig. 4f). Together, these results therefore demonstrated direct correlation between impaired recruitment to Complex I, defective M1-ubiquitylation in Complex I and cell death induction. In addition, these results also demonstrated that the protective role of A20 is mediated by its ZF7 domain, independently of the functions attributed to its ZF4 and OTU domains.

**Fig. 5:**
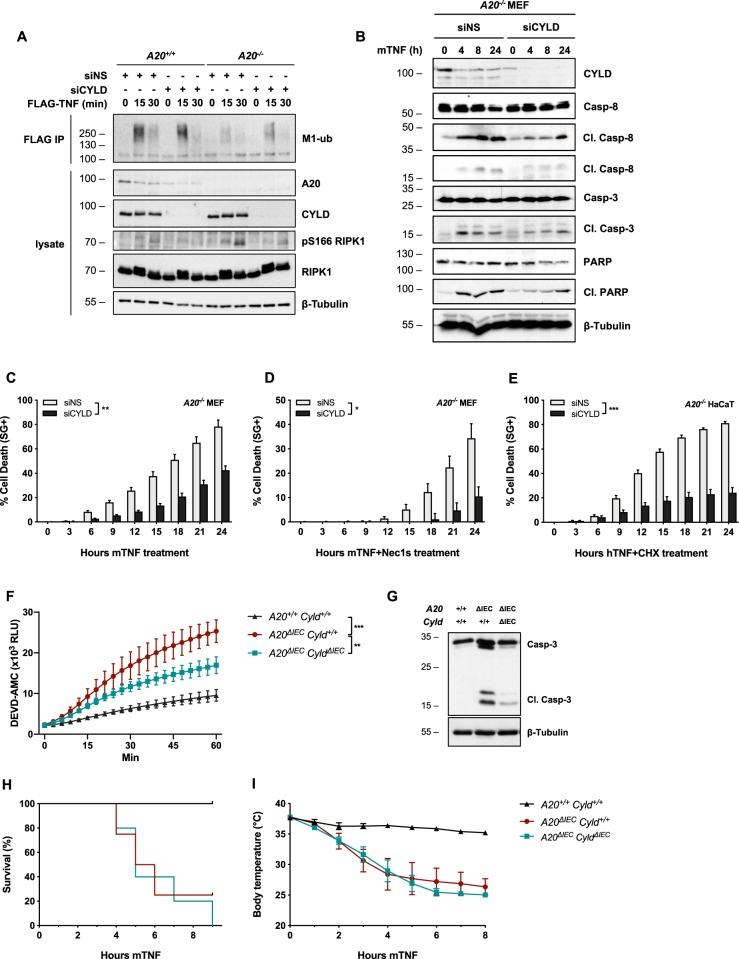
CYLD depletion rescues defective M1-ubiquitylation in Complex I and consequently protects A20-deficient cells from TNF-induced apoptosis. **a** *A20*^*+/+*^ and *A20*^*−/−*^ MEFs were transfected with siRNA targeting CYLD or non-specific siRNA (NS) and stimulated with 1 µg/ml FLAG-hTNF for the indicated duration. TNFR1 Complex I was subsequently FLAG-immunoprecipitated. Protein levels were determined by immunoblotting. **b**–**d** *A20*^*−/−*^ MEFs were transfected with siRNA targeting CYLD or nonspecific siRNA (NS). Cells were pretreated with the indicated compounds for 30 min before stimulation with 10 ng/ml mTNF. Cell death markers were monitored by immunoblotting (**b**) and cell death was measured in function of time by SytoxGreen (SG) positivity (**c**, **d**). **e** *A20*^*−/−*^ HaCaTs were transfected with siRNA targeting CYLD or nonspecific siRNA (NS). Cells were pretreated with CHX for 30 min before stimulation with 10 ng/ml hTNF. Cell death was measured in function of time by SytoxGreen (SG) positivity. **f**, **g** *A20*^*+/+*^ *Cyld*^*+/+*^ (*n* = 3), *A20*^*ΔIEC*^ *Cyld*^*+/+*^ (*n* = 3), and *A20*^*ΔIEC*^ *Cyld* ^*ΔIEC*^ (*n* = 3) were injected i.p. with 5 µg/20 g mTNF. Caspase activity was quantified from small intestinal tissue homogenates 2 h post injection using a fluorescent caspase-activity probe (DEVD-AMC) (**f**) or via immunoblotting (**g**). **h**, **i** *A20*^*+/+*^ *Cyld*^*+/+*^ (*n* = 5), *A20*^*ΔIEC*^ *Cyld*^*+/+*^ (*n* = 4), and *A20*^*ΔIEC*^ *Cyld* ^*ΔIEC*^ (*n* = 5) were injected i.p. with 5 µg/20 g mTNF. Cumulative survival rates (**h**) and body temperature (**i**) were determined in function of time. Temperatures are presented as mean ± SEM. Statistical significance for body temperatures of the mice was determined using two-way ANOVA followed by a Tukey post hoc test. Survival curves were compared using log-rank Mantel–Cox test. Cell death experiments are presented as mean ± SEM of three independent experiments. Statistical significance for the cell death assays was determined using two-way ANOVA followed by a Tukey post hoc test. Significance between samples is indicated in the figure as follows: *p < 0.05; **p < 0.01; ***p < 0.001; ns non significant.

We found that, similar to *A20*^*−/−*^ MEFs, cells deprived of linear ubiquitylation by HOIP deletion also succumbed to single TNF stimulation by RIPK1 kinase-dependent and -independent apoptosis (Fig. S4B, C), further supporting the idea that altered M1-ubiquitylation in Complex I drives apoptosis in A20-deficient cells. To challenge this hypothesis, we evaluated the effect of restoring M1-ubiquitylation in *A20*^*−/−*^ MEFs by repressing CYLD, a DUB known to hydrolyze K63- and M1-ubiquitin chains in the TNFR1 pathway^24,44^. Repressing CYLD in *A20*^*−/*^^*−*^ MEFs partially restored M1-ubiquitylation in Complex I (Fig. 5a), reduced cytosolic RIPK1 autophosphorylation on S166 (Fig. 5a), and rescued A20-deficient MEFs and HaCaTs from TNF-induced RIPK1 kinase-dependent and -independent apoptosis (Fig. 5b–e). In contrast, CYLD depletion failed to rescue HOIP-deficient cells from TNF-induced death, supporting an M1-ubiquitin-specific effect of CYLD depletion in *A20*^*–/–*^ MEFs (Fig. S5A). To evaluate if the in vitro protective effect observed by CYLD deletion in *A20*^*−/*^^−^ cells could be translated in vivo, we generated *A20*^*ΔIEC*^ *Cyld*^*ΔIEC*^ mice^45^. These mice were significantly protected from TNF-induced intestinal apoptosis when compared to *A20*^*ΔIEC*^ mice, as shown by reduced caspase-3 processing and activity in small intestinal homogenates (Fig. 5f, g). Nevertheless, *A20*^*ΔIEC*^ and *A20*^*ΔIEC*^ *Cyld*^*ΔIEC*^ mice responded similarly to TNF-induced hypothermia and lethality (Fig. 5h, i), which could potentially be explained by the cell death-independent excessive inflammation resulting from the combined removal of these DUBs. Together, our results support a model in which constitutively expressed A20 protects cells from TNF-induced RIPK1 kinase-dependent and -independent apoptosis by antagonizing the CYLD-mediated degradation of the linear ubiquitin network associated to Complex I.

### A20 additionally protects cells from TNF-induced death independently of linear ubiquitylation

**Fig. 6:**
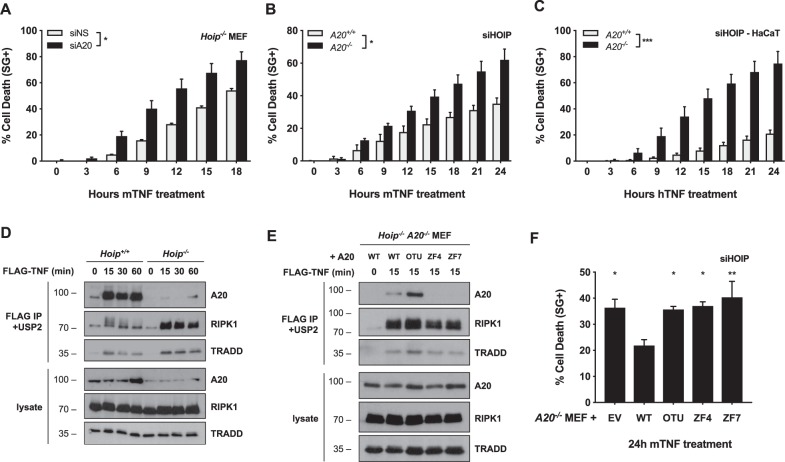
A20 additionally protects cells from death through its deubiquitylase activity. **a** *Hoip*^*−/−*^ MEFs were transfected with siRNA targeting A20 or nonspecific siRNA (NS). Cells were stimulated with 10 ng/ml mTNF and cell death was measured in function of time by SytoxGreen (SG) positivity. **b** *A20*^*+/+*^ and *A20*^*−/−*^ MEFs were transfected with siRNA targeting HOIP. Cells were stimulated with 10 ng/ml mTNF and cell death was measured in function of time by SytoxGreen (SG) positivity. **c** *A20*^*+/+*^ and *A20*^*−/−*^ HaCaTs were transfected with siRNA targeting HOIP. Cells were stimulated with 10 ng/ml hTNF and cell death was measured in function of time by SytoxGreen (SG) positivity. **d** *Hoip*^*+/+*^ and *Hoip*^*−/−*^ MEFs were stimulated with 1 µg/ml FLAG-hTNF for the indicated duration. TNFR1 Complex I was FLAG-immunoprecipitated, followed by USP2 treatment on the post-IPs. Protein levels were determined by immunoblotting. **e** *Hoip*^−/−^ *A20*^*−/−*^ MEFs lentivirally reconstituted with wild-type A20, empty vector (EV) or indicated A20 mutants were stimulated with 1 µg/ml FLAG-hTNF for the indicated duration. TNFR1 Complex I was subsequently FLAG-immunoprecipitated, followed by USP2 treatment on the post-IPs. Protein levels were determined by immunoblotting. **f** *A20*^*−/−*^ MEFs lentivirally reconstituted with wild-type A20, empty vector (EV) or indicated A20 mutants were transfected with siRNA targeting HOIP. Cells were stimulated with 10 pg/ml mTNF and cell death was measured in function of time by SytoxGreen (SG) positivity. Statistical significance for the cell death assays was determined using two-way ANOVA followed by a Tukey post hoc test (**a**–**c**) or using one-way ANOVA followed by a Dunnett post hoc test (**f**). Significance between samples is indicated in the figure as follows: *p < 0.05; **p < 0.01; ***p < 0.001; ns non significant.

To determine if A20 additionally protects cells by a mechanism independent of M1-ubiquitylation, we repressed A20 in HOIP-depleted cells. Remarkably, A20 inactivation further sensitized HOIP-depleted MEFs and HaCaT cells to TNF-induced death (Fig. 6a–c). In line with a protective function of A20 at the level of Complex I, we found that A20 was still recruited to Complex I early after TNF stimulation in *Hoip*^*−/−*^ MEFs, although to a much lower extent than in control MEFs (Fig. 6d). In HOIP-proficient cells, recruitment of A20 to Complex I was solely depending on its ZF7 (Fig. 4f), which was in accordance with previous studies reporting specific binding of ZF7 to linear ubiquitin chains^24,26,37^. In HOIP-deficient cells, we instead found that A20's recruitment to Complex I became dependent on its ZF4, but surprisingly also remained dependent on its ZF7 (Fig. 6e). Indeed, the ZF7 mutant, which still has an intact ZF4, was also unable to associate with Complex I. These results indicated that, in absence of linear ubiquitylation, the ZF4 and ZF7 domains of A20 cooperate to bind to other types of ubiquitin chains associated to Complex I, potentially K63-linked chains. In accordance with the notion that A20 exerts its antideath role in Complex I, re-expressing WT A20, but not the ZF4 or ZF7 mutant, provided protection to TNF-induced death in MEFs simultaneously depleted of HOIP and A20 (Fig. 6f). Interestingly, the OTU mutant also failed to provide protection in these HOIP-deficient cells, despite the fact that it was efficiently recruited to Complex I (Fig. 6e, f). Since the OTU domain was not required for A20's antideath function in HOIP-proficient cells (Fig. 4e), these results indicate a switch in the molecular mechanism employed by A20 to protect the cells from death in conditions of defective M1-ubiquitylation.

**Fig. 7:**
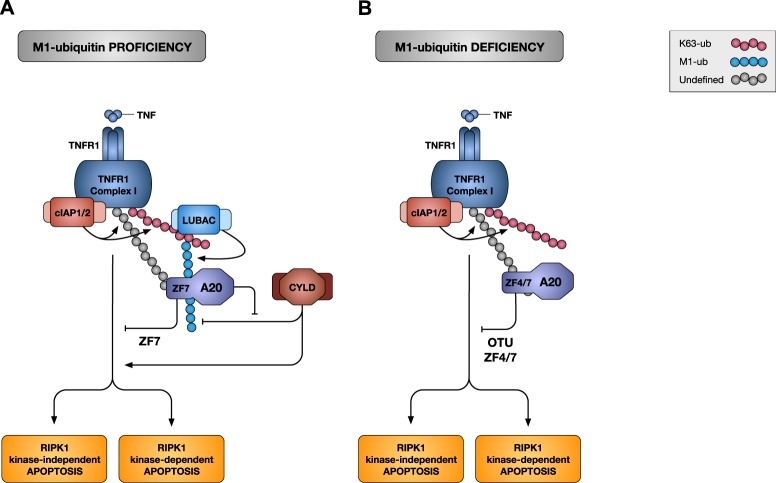
Schematic overview of the prosurvival functions of A20. **a** M1-ubiquitylation-proficient cells: A20 protects cells from TNF-mediated apoptosis by antagonizing CYLD-mediated degradation of M1-ubiquitin chains. Binding of TNF to TNFR1 induces the formation of TNFR1 Complex I. The E3 ubiquitin ligases cIAP1/2 conjugate Complex I components with ubiquitin chains of different types, including K63-linked chains. The multiprotein E3 complex LUBAC docks on these newly formed K63-linked chains to further conjugate Complex I components with M1-linked (linear) ubiquitin chains; creating, in some cases, hybrid K63/M1 chains. Within minutes of TNF sensing, constitutively expressed A20 is recruited to Complex I via the binding of its ZF7 domain to ubiquitin chains of different types, including M1-linked chains. Recruited A20 promotes cell survival by antagonizing CYLD-mediated hydrolysis of the M1-linked ubiquitin chains in the complex. In conditions of A20 deficiency, CYLD dismantles the M1-linked ubiquitin network, ultimately inducing RIPK1 kinase-dependent and -independent apoptosis. **b** In absence of M1-ubiquitylation: A20 protects cells from TNF-induced apoptosis by deubiquitylating a yet to be identified substrate. Constitutively expressed A20 is still recruited to Complex I in absence of M1-ubiquitylation through ZF4/ZF7-mediated binding to the residual ubiquitin chains, potentially the K63-linked chains. Recruited A20 limits the extent of TNF-induced apoptosis by deubiquitylating unknown substrate(s). The DUB activity of A20 may counteract the K48-ubiquitin-mediated proteasomal degradation of a prosurvival molecule and/or the K63-ubiquitin-dependent stabilization of a prodeath signaling platform.

Taken together, our results demonstrate that in normal conditions, constitutively expressed A20 protects the cells from TNF-induced apoptosis by ZF7-dependent binding to the linear ubiquitin chains associated to Complex I, and thereby protecting them from CYLD-mediated degradation. In contrast, in absence of M1-ubiquitylation, the protective role of A20 relies on its ZF4/ZF7-dependent recruitment to Complex I, and on the deployment of its deubiquitylase activity (Fig. 7a, b).

## Discussion

In this study, we characterized the prosurvival role of A20 in cells exposed to TNF, and evaluated some of our in vitro findings in an in vivo model of lethal inflammation induced by the administration of low doses of TNF to mice specifically lacking A20 in IECs (*A20*^*ΔIEC*^)^39^. We found that A20 deficiency suffices to sensitize MEFs and intestinal organoids to TNF-induced death in the absence of any additional inhibitor of the TNFR1 pathway. We found that A20 deficiency results in the simultaneous occurrence of RIPK1 kinase-dependent and -independent apoptosis, but not in the spontaneous induction of necroptosis. In line with these in vitro and ex vivo results, genetic inhibition of RIPK1 kinase activity, but not RIPK3 deficiency, significantly delayed the lethality of *A20*^*ΔIEC*^ mice injected with TNF. These results demonstrate the contribution of RIPK1 kinase-dependent apoptosis, and not necroptosis, to the lethal inflammatory phenotype of *A20*^*ΔIEC*^ mice exposed to TNF. RIPK1 kinase-dependent cell death is reported to drive the pathogenesis of several inflammatory diseases in the mouse, and RIPK1 inhibitors are now in clinical trials for the treatment of inflammatory bowel disease (IBD), psoriasis, and rheumatoid arthritis^46^. The fact that the *A20*^*ΔIEC*^ *Ripk1*^*D138N/D138N*^ mice still died at a later stage in our experiment may limit the enthusiasm for the potential use of RIPK1 inhibitors in IBD patients harboring *A20* mutations. The lethality of the *A20*^*ΔIEC*^ *Ripk1*^*D138N/D138N*^ mice can potentially be explained by the remaining occurrence of RIPK1 kinase-independent apoptosis of IECs, but also by the excessive NF-κB signaling originating from A20 deficiency.

The TNF-mediated NF-κB-dependent upregulation of A20 is widely considered as an inhibitory feedback mechanism to shut down NF-κB activation. An accepted model is that upregulated A20 is recruited to TNFR1 Complex I to mediate its inhibitory role on NF-κB activation. Our results show that constitutively expressed A20 is also recruited to the TNFR1 Complex I within minutes of TNF sensing; thus, far before the pool of de novo synthesized A20. Importantly, we show that the pool of constitutively expressed A20 protects cells from TNF-induced death independently of the upregulated A20. This may indicate that two different pools of A20 respectively regulate cell death and NF-κB activation, thereby uncoupling these two functions of A20. We can however not exclude the possibility that the de novo synthesized A20 also contributes to A20's prosurvival function.

We show that association of constitutively expressed A20 to Complex I occurs in a cIAP1/2-dependent manner and is solely mediated by A20's ZF7 domain under HOIP-proficient conditions. In line with a previous report^24^, we found that removal of A20, or preventing its recruitment to Complex I by mutating its ZF7, results in increased cell death, and is associated with increased K63-ubiquitylation and decreased M1-ubiquitylation of Complex I components. We now demonstrate that the defective M1-ubiquitylation of Complex I is responsible for the induction of RIPK1 kinase-dependent and -independent apoptosis in A20-deficient cells. Indeed, we first excluded the increase in K63-ubiquitylation as a driver of cell death by showing that mutation in A20's ZF4 or OTU domains also resulted in elevated K63-ubiquitylation but without sensitizing the cells to death. Those results also demonstrated that the E3 ubiquitin ligase and deubiquitylase activities of A20 are not required to protect these cells from death. Then, by deleting CYLD in A20-deficient cells, we were able to partially restore the M1-linked ubiquitin chains and revert the sensitivity towards TNF-mediated cell death, thereby demonstrating that reduced M1-ubiquitylation of Complex I causes death in A20-deficient cells. We found that IEC-specific CYLD deletion protected *A20*^*ΔIEC*^ mice from TNF-induced intestinal apoptosis, indicating that this mechanism also occurs in vivo. However, we did not observe reduced or delayed lethality of *A20*^*ΔIEC*^ *Cyld*^*ΔIEC*^ mice upon TNF injection. It is important to note that *A20*^*ΔIEC*^ *Cyld*^*ΔIEC*^ mice lack two potent NF-κB repressors and, consequently, TNF administration to these mice may trigger massive NF-κB-dependent inflammation that drives the observed lethality. Together, our results highlight a complex interplay between A20 and CYLD in regulating life-death decisions by finetuning M1-linked ubiquitin chains in TNFR1 Complex I (Fig. 7a).

Our data reveals that Complex I-associated M1-ubiquitin chains regulate both RIPK1 kinase-dependent and -independent death. Linear ubiquitylation-dependent phosphorylation of RIPK1 by IKKα/β and TBK1/IKKε was recently reported to repress RIPK1 activation and RIPK1 kinase-dependent apoptosis^9,10,14,47^. Accordingly, we found that the reduced M1-ubiquitylation caused by A20 deficiency activates RIPK1 and triggers RIPK1 kinase-dependent apoptosis. How defective M1-ubiquitylation additionally promotes RIPK1-independent apoptosis is less clear. The NF-κB-dependent upregulation of prosurvival proteins is known to protects cells from RIPK1-independent apoptosis^15,43^. In light of the reported role of linear ubiquitylation as docking site for NEMO-IKKα/β activation, it would be easy to explain the induction of RIPK1-independent apoptosis by a defect in NF-κB signaling. The problem resides on the fact that A20-deficiency instead leads to sustained NF-κB activation. As a potential alternative mechanism, we can envision that reduced M1-ubiquitylation destabilizes Complex I and thereby facilitates dissociation of its components for their subsequent association into the RIPK1-independent apoptosis-inducing Complex IIa. The discrepancy between reduced M1-ubiquitylation of Complex I and the sustained NF-κB activation observed in absence of A20 may instead be explained by the increase in K63-ubiquitylation, which would somehow compensate for the missing M1-ubiquitin linkages.

We found that the ability of A20 to protect cells from death is not limited to antagonizing the degradation of M1-ubiquitin chains by CYLD. Indeed, A20 is still recruited to Complex I in cells lacking M1-ubiquitylation, where it acts as a molecular brake limiting the extent of cell death by deploying its DUB activity (Fig. 7b). In M1-proficient cells, recruitment of A20 to Complex I was solely depending on its ZF7, known to preferentially bind M1-ubiquitin chains^26,28^. In absence of M1-ubiquitylation, recruitment of A20 to Complex I became dependent on its ZF4 and ZF7. These results indicate that the ZF7 domain, unlike widely assumed, does not exclusively bind M1-ubiquitin chains, and that the ZF4 and ZF7 can cooperate in binding to other types of ubiquitin chains associated to Complex I, potentially K63-linked chains. We found that both the ZF4 and ZF7 domains of A20 were required to provide protection to TNF-induced death in HOIP-depleted MEFs, indicating that A20 also exert its anti-death role in Complex I in these cells. Interestingly, the requirement of A20's DUB activity to protect cells from death was only observed in M1-ubiquitylation-deficient cells. This indicates a switch in the molecular mechanism employed by A20 to protect the cells in these two different conditions. In M1-proficient cells, A20's prosurvival function consists in antagonizing CYLD-mediated degradation of M1-ubiquitin chains, potentially just by binding to the linear chains via its ZF7 domain, as previously suggested^24,26,37^. In absence of M1-ubiquitylation, recruitment of A20 to Complex I limits the extent of cell death by deubiquitylating yet to be identified substrate(s). Since A20 was reported to possess DUB activity towards K48- and K63-ubiquitin linkages^21–23^, this additional prosurvival role may counteract the K48-ubiquitin-mediated proteasomal degradation of a prosurvival molecule and/or the K63-ubiquitin-dependent stabilization of a prodeath signaling platform. Identifying the substrate(s) of A20's DUB activity in this context therefore represents an exciting challenge.

Together, our results demonstrate two distinct molecular mechanisms by which constitutively expressed A20 protect cells from TNF-induced apoptosis, thereby providing new insights on the complexity of this potent anti-inflammatory molecule.

## Materials and methods

### Antibodies and reagents

The following antibodies were used throughout this manuscript: anti-A20 (Santa Cruz sc-166692, 1:1000), anti-RIPK1 (BD Biosciences #610459, 1:2000; Cell Signaling #3493, 1:2000), anti-IκBα (Santa Cruz sc-371, 1:2000), anti-β-tubulin-HRP (Abcam ab21058, 1:10000), anti-phospho Ser166 RIPK1 (Cell Signaling #31122, 1:1000), anti-cleaved caspase-8 (Cell Signaling #9429, 1:1000), anti-caspase-8 (Abnova MAB3429, 1:1000), anti-caspase-3 (Cell Signaling #9662, 1:1000), anti-PARP (Cell Signaling # 9532S, 1:1000), anti-cleaved PARP (Cell Signaling #9544S, 1:1000), anti-phospho Ser345 MLKL (Millipore MABC1158, 1:1000), anti-MLKL (Millipore MABC604, 1:1000), anti-TRADD (Bio-Rad AHP2533, 1:1000), anti-RIPK3 (ProSci #2283, 1:3000), anti-CYLD (Santa Cruz sc-74435, 1:500), ant-cIAP1 (Enzo Life Science ALX-803-335-C100, 1:1000), anti-TNFR1 (Cell Signaling #13377, 1:1000), anti-HOIP (Abcam ab46322, 1:1000), anti-M1-ubiquitin (1E3 Merck MABS199, LUB9 Merck MABS451, 1:1000), and anti-K63-ubiquitin (Apu3 Merck #05-1308). Recombinant mouse and human TNF-α (concentration indicated in the figure legends) and FLAG-tagged hTNF (1 μg/ml) were purchased from the VIB Protein Service Facility (Ghent, Belgium). Rat recombinant untagged USP2 was purchased from Enzo Life Sciences (BML-UW9850-0100). Lambda protein phosphatase was obtained from New England Biolabs (P0753s). The following compounds were used: IKKi (TPCA-1, 5 μM, Tocris Bioscience), zVAD-fmk (50 μM, Bachem), CHX (2 µg/ml, Sigma-Aldrich), TBKi (MRT67307, 2 µM, Bioconnect; GSK8612, 10 µM, MedChemExpress), TAK1i (5Z-7-Oxozeaenol, 500 nM, Analyticon Discovery), BV6 (1 μM, Selleckchem), Staurosporin (2 µM, Sigma), Etoposide (10 µM, Enzo Life Sciences), ML-162 (10 µM, Aobious Inc), Tunicamycin (10 µg/ml, Sigma), and Doxycycline (1 µg/ml, Sigma). Nec-1s (UAMC-02197) was produced by the Laboratory of Medicinal Chemistry (University of Antwerp, Belgium).

### Mice

*A20*^*ΔIEC*^, *Ripk1*^*D138N/D138N*^, and *Cyld*^*fl/fl*^ mice were described earlier^39,40,45^. *A20*^*ΔIEC*^ *Cyld*^*ΔIEC*^ mice were generated by crossing the *Cyld*^*fl/fl*^ mice with the *A20*^*ΔIEC*^ mice (expressing Cre under the Villin promotor), ultimately generating mice specifically lacking A20 and CYLD in intestinal epithelial cells. All crosses were validated by genotyping. Primary cultures were isolated from littermate mice.

### TNF shock model

All experiments on mice were conducted according to institutional, national and European animal regulations. Animal protocols were approved by the ethical committee of Ghent University. For the TNF shock model, 5 μg/20 g mTNF (diluted in endotoxin-free phosphate-buffered saline (PBS) (pH 6.8)) was intraperitoneally (i.p.) injected. Mortality and body temperature were monitored until 24 h after mTNF injection. Rectal body temperature was recorded with an industrial electric thermometer (Comark Electronics, Norwich, UK; model 2001). Dead mice were considered to be at 25 °C.

### Cell lines and primary cultures

Mouse embryonic fibroblasts (MEFs) and mouse dermal fibroblasts were cultured in Dulbecco’s modified Eagle’s medium supplemented with 10% fetal calf serum, l-glutamine (200 mM) and sodium pyruvate (400 mM) in normoxic conditions (5% CO_2_). When the cells were still at the primary state, 0.1% β-mercaptoethanol and 100 ng/ml penicillin/streptomycin was added to the medium. Primary *A20*^*+/+*^ and *A20*^*−/−*^ MEFs were isolated from E12.5 littermate embryos following standard protocol and cultured under low-oxygen conditions (3% O_2_). MEFs were subsequently immortalized by an SV40-containing construct with JetPRIME (Polyplus transfection) according the manufacturer’s instructions. *Hoip*^*−/−*^ *Tnf*^*−/−*^ and *Hoip*^*+/+*^ *Tnf*^*−/−*^ MEFs were a kind gift from Prof. H. Walczak. *Hoip*^*−/−*^ *A20*^*−/*^^*−*^ *Tnf*^*−/−*^ MEF cells were generated using CRISPR/Cas9. In short, *Hoip*^*−/−*^ *Tnf*^*−/−*^ MEF cells were transfected with a plasmid encoding Cas9 nuclease and a guide sequence targeting mouse A20 (GGAGCTTGTCAGTACATGTG), subcloned and screened for A20 deficiency. Human immortalized keratinocytes (HaCaTs) were cultured in Dulbecco’s modified Eagle’s medium supplemented with 10% fetal calf serum and l-glutamine (200 mM). A20-deficient HaCaT cells were generated using CRISPR/Cas9. In short, WT HaCaT cells were transfected with a plasmid encoding Cas9 nuclease and a guide sequence targeting human A20 (TGGATGATCTCCCGAAACTG), subcloned and screened for A20 deficiency. Intestinal organoids were derived from small intestine as previously described^48^. Briefly, a 5-cm piece of duodenum/jejunum was dissected and washed in PBS. The intestine was opened longitudinally, villi were scraped away, and the tissue was chopped into pieces of 2–3 mm. After thorough washing in PBS, the pieces were incubated in 2 mM EDTA/PBS for 30 min at 4 °C on a rocking platform. The mixture was passed through a 70-µm cell strainer, and crypt fractions were isolated and purified by successive centrifugation steps. One milliliter of Matrigel (BD Biosciences) was added to a pellet of 100–1000 crypts, and 50-µl drops of crypt-containing Matrigel were added to prewarmed wells in a 24-well plate. After polymerization, 500 µl complete growth medium containing EGF (Peprotech), R-Spondin1 (R&D), and Noggin (Peprotech) was added and refreshed every 2 days.

### RNA interference

MEFs were seeded at 1 × 10^5^ cells per well in 6-well plates. The cells were transfected 24 h later with specific siRNAs using Dharmafect1 (Dharmacon) according to the manufacturer’s instructions. Cells were reseeded in appropriate plates 24 h post transfection and analyzed 48 h post transfection. For silencing genes in HaCaTs, 3000 cells were seeded per well in triplicates in a 96-well plate. The cells were immediately transfected with specific siRNA using INTERFERin (Polyplus transfection) according to the manufacturer’s instructions. Medium was changed 24 h post transfection and cells were analyzed 48 h post transfection. All siRNA’s were purchased from Dharmacon (ON-TARGETplus SMARTpool), except siRNA targeting mouse A20 (Ambion).

### Cell death assays

For cell death assays in MEFs, cells were seeded the day before at 10,000 per well in triplicates in a 96-well plate. Cells reconstituted with A20 mutants were treated with 1 µg/ml doxycycline 24 h prior to stimulation. The next day, cells were pretreated with the indicated compounds for 30 min and then stimulated with the indicated concentration of mTNF in the presence of 2,5 μM SytoxGreen (Invitrogen) and 20 μM Ac-DEVD-MCA (PeptaNova). SytoxGreen intensity and caspase-3 activation were measured at intervals of 1 h using a Fluostar Omega fluorescence plate reader, with an excitation filter of 485 nm (SytoxGreen) or 360 nm (Ac-DEVD-MCA), an emission filter of 520 nm (SytoxGreen) or 460 nm (Ac-DEVD-MCA), gains set at 1100, 20 flashes per well and orbital averaging with a diameter of 3 mm. Percentage of cell death was calculated as (induced fluorescence − background fluorescence)/(maximum fluorescence − background fluorescence) × 100. The maximal fluorescence is obtained by full permeabilization of the cells by using Triton X-100 at a final concentration of 0.1%. All cell death and caspase-3 activation data are presented as mean ± SEM of *n* (indicated in the figure) independent experiments, unless stated otherwise. For intracellular caspase-activity measurement, cells were seeded the day before at 10,000 per well in triplicates in a 96-well plate. The next day, cells were pretreated with the indicated compounds for 30 min and then stimulated with the indicated concentration of mTNF. Cells were lysed in caspase lysis buffer (1% NP-40, 200 mM NaCl, 10 mM Tris-HCl pH 7, 5 mM EDTA, 10% glycerol, freshly supplemented with 1 mM leupeptin, 0.1 mM aprotinin and 1 mM PMSF) and volume was adjusted to reach a protein concentration of 2 µg/µl. A 20 µg of cell lysate was added to 140 µl of CFS buffer (10 mM HEPES pH 7.5, 220 mM mannitol, 68 mM sucrose, 2 mM NaCl, 2 mM MgCl2, 2.5 mM KH2PO4, freshly supplemented with 10 mM DTT) containing 50 µM Ac-DEVD-MCA (PeptaNova). Caspase-3 activation was measured at intervals of 5 min using a Fluostar Omega fluorescence plate reader, as described above. To measure cell death in HaCaTs, cells were seeded the day before at 10,000 per well in triplicates in a 96-well plate. The next day, cells were pretreated with the indicated compounds for 30 min and then stimulated with the indicated concentration of hTNF in the presence of 1 μM SytoxGreen (Invitrogen). SytoxGreen (SG) positivity was measured at intervals of 1 h using an Essen BioScience IncuCyte. Percentage of cell death was calculated as (number of SG + cells/mm^2^ − number of unstimulated SG + cells/mm^2^)/(maximum number of SG + cells/mm^2^ − number of unstimulated SG + cells/mm^2^) × 100. To measure cell death in organoid cultures, primary organoids were isolated and plated in triplicates in a 96-well plate. Five days later, organoids were pretreated with the indicated compounds for 30 min and then stimulated with the indicated concentration of mTNF in the presence of 3 μM propidium iodide (PI) (Sigma) and 1.6 μM Hoechst (Invitrogen). Cells were imaged at intervals of 1 h using a PerkinElmer Operetta High-Content Imaging System. Organoids were defined based on size, shape and Hoechst positivity. Cell death was calculated by mean PI intensity per organoid. To analyze cell death by FACS, 3 × 10^5^ cells were seeded in a 21 cm^2^ petridish per condition. The next day, cells were pretreated with the indicated compounds for 30 min and then stimulated with the indicated concentration of mTNF. After stimulation, cells + supernatant was collected, washed and stained with DRAQ7 (500 nM, Biostatus) and CellEvent Caspase-3/7 Green activity probe (500 nM, Life Technologies) for 45′ at RT. Cell death stainings were measured using a 752 LP filter (DRAQ7) and 507 LP filter (Caspase-3/7 Green) on the FACSVerse (BD).

### Production of GST-UBAN

Recombinant GST-UBAN were produced in BL21(DE3) cells. In brief, BL21(DE3) cells were transformed with the plasmid encoding GST-UBAN and protein expression was induced with 0.5 M IPTG. After 4 h, cells were collected and lysed in lysis buffer (20 mM Tris-HCl pH 7.5, 10 mM EDTA, 5 mM EGTA, 150 mM NaCl, 1 mM DTT supplemented with phosphatase and protease inhibitor cocktail tablets (Roche Diagnostics)), sonicated and cleared by centrifugation. After centrifugation, Triton-X100 (0.5% final concentration) was added to the supernatant, which was then transferred onto prewashed glutathione beads and left rotating for 2 h at 4 °C. After incubation, the beads were centrifuged, washed twice with washing buffer (20 mM Tris-HCl pH 7.5, 10 mM EDTA, 150 mM NaCl, 0.5% Triton-X100) and resuspended in resuspension buffer (20 mM Tris-HCl pH 7.5, 0.1% β-mercaptoethanol, 0.05% sodiumazide), ready to be used.

### Immunoprecipitation

TNFR1 Complex I was isolated as described^49^. In brief, 8 × 10^6^ cells were seeded in a 150 cm^2^ petridish per condition for both TNFR1 complex I (CI IP) and M1-ubiquitin (UBAN IP) IPs. Cells reconstituted with A20 mutants were treated with 1 µg/ml doxycycline 24 h prior to stimulation. Cells were pretreated as indicated in the figure legends and subsequently cells stimulated (or not) with 1 μg/ml FLAG-hTNF (CI IP) or 1 μg/ml hTNF (UBßAN IP). Cells were then washed 2 times in ice-cold PBS and lysed in 1 ml NP-40 lysis buffer (10% glycerol, 1% NP-40, 150 mM NaCl and 10 mM Tris-HCl pH 8 supplemented with phosphatase and protease inhibitor cocktail tablets (Roche Diagnostics)) (C1) or 1 ml RIPA lysis buffer (150 mM NaCl, 1% NP-40, 0.5% sodium deoxycholate, 0.1% SDS, 10 mM Tris-HCl pH 8 supplemented with phosphatase and protease inhibitor cocktail tablets (Roche Diagnostics)) (UBAN IP). The cell lysates were cleared by centrifugation for 15 min at 4 °C and the supernatants were then incubated overnight with FLAG M2 affinity gel (Sigma-Aldrich) for CI IPs, or with GST-UBAN-containing glutathione beads (UBAN IP). The next day, the beads were washed three times in NP-40 (C1) or RIPA (UBAN IP) lysis buffer. For C1 IPs, the proteins were eluted from the beads using 200 ng/ml 3× FLAG peptide. Lysate or beads were then resuspended in 60 μL 1× laemmli buffer for direct analysis. However, when indicated, protein complexes were additionally deubiquitylated (by USP2 treatment) and dephosphorylated (by lambda protein phosphatase treatment) post-IP. To do so, beads were resuspended after the final wash step in 50 μL 1× DUB/λPP buffer (50 mM Tris-HCl pH8, 50 mM NaCl, 5 mM DTT and 1 mM MnCl_2_). Subsequently, 1.2 μg USP2 (Enzo Life Sciences) and 400U λ PPase were added when indicated. Enzymatic reactions were allowed to proceed for 30 min at 37 °C and subsequently quenched by the addition of 12.5 μl 5× laemmli buffer. All IPs were analyzed by immunoblotting.

## Supplementary information

Supplementary Figure 1

Supplementary Figure 2

Supplementary Figure 3

Supplementary Figure 4

Supplementary Figure 5

## References

[1] Lis K, Kuzawińska O, Bałkowiec-Iskra E (2014). Tumor necrosis factor inhibitors—state of knowledge. Arch. Med. Sci..

[2] Haas TL (2009). Recruitment of the linear ubiquitin chain assembly complex stabilizes the TNF-R1 signaling complex and is required for TNF-mediated gene induction. Mol. Cell.

[3] Bertrand MJM (2008). cIAP1 and cIAP2 facilitate cancer cell survival by functioning as E3 ligases that promote RIP1 ubiquitination. Mol. Cell.

[4] Bertrand MJM (2011). cIAP1/2 are direct E3 ligases conjugating diverse types of ubiquitin chains to receptor interacting proteins kinases 1 to 4 (RIP1–4). PLoS ONE.

[5] Kanayama A (2004). TAB2 and TAB3 activate the NF-κB pathway through binding to polyubiquitin chains. Mol. Cell.

[6] Emmerich Christoph H., Bakshi Siddharth, Kelsall Ian R., Ortiz-Guerrero Juanma, Shpiro Natalia, Cohen Philip (2016). Lys63/Met1-hybrid ubiquitin chains are commonly formed during the activation of innate immune signalling. Biochemical and Biophysical Research Communications.

[7] Rahighi S (2009). Specific recognition of linear ubiquitin chains by NEMO is important for NF-kappaB activation. Cell.

[8] Clark K (2011). Novel cross-talk within the IKK family controls innate immunity. Biochem. J..

[9] Lafont E (2018). TBK1 and IKKε prevent TNF-induced cell death by RIPK1 phosphorylation. Nat. Cell Biol..

[10] Xu D (2018). TBK1 suppresses RIPK1-driven apoptosis and inflammation during development and in aging. Cell.

[11] Lork Marie, Verhelst Kelly, Beyaert Rudi (2017). CYLD, A20 and OTULIN deubiquitinases in NF-κB signaling and cell death: so similar, yet so different. Cell Death & Differentiation.

[12] Annibaldi A, Meier P (2018). Checkpoints in TNF-induced cell death: implications in inflammation and cancer. Trends Mol. Med..

[13] Ting AT, Bertrand MJM (2016). More to life than NF-κB in TNFR1 signaling. Trends Immunol..

[14] Dondelinger Y (2015). NF-κB-independent role of IKKα/IKKβ in preventing RIPK1 kinase-dependent apoptotic and necroptotic cell death during TNF signaling. Mol. Cell.

[15] Wang L, Du F, Wang X (2008). TNF-α induces two distinct caspase-8 activation pathways. Cell.

[16] Catrysse L, Vereecke L, Beyaert R, van Loo G (2014). A20 in inflammation and autoimmunity. Trends Immunol..

[17] Ma A, Malynn BA (2012). A20: linking a complex regulator of ubiquitylation to immunity and human disease. Nat. Rev. Immunol..

[18] Opipari AW, Hu HM, Yabkowitz R, Dixit VM (1992). The A20 zinc finger protein protects cells from tumor necrosis factor cytotoxicity. J. Biol. Chem..

[19] Cooper JT (1996). A20 blocks endothelial cell activation through a NF-κB-dependent Mechanism. J. Biol. Chem..

[20] Tewari M (1995). Lymphoid expression and regulation of A20, an inhibitor of programmed cell death. J. Immunol..

[21] Evans PC (2004). Zinc-finger protein A20, a regulator of inflammation and cell survival, has de-ubiquitinating activity. Biochem. J..

[22] Komander D, Barford D (2008). Structure of the A20 OTU domain and mechanistic insights into deubiquitination. Biochem. J..

[23] Lin S-C (2008). Molecular basis for the unique deubiquitinating activity of the NF-κB inhibitor A20. J. Mol. Biol..

[24] Draber P (2015). LUBAC-recruited CYLD and A20 regulate gene activation and cell death by exerting opposing effects on linear ubiquitin in signaling complexes. Cell Rep..

[25] Lu TT (2013). Dimerization and ubiquitin mediated recruitment of A20, a complex deubiquitinating enzyme. Immunity.

[26] Verhelst K (2012). A20 inhibits LUBAC‐mediated NF‐κB activation by binding linear polyubiquitin chains via its zinc finger 7. EMBO J..

[27] Wertz IE (2015). Phosphorylation and linear ubiquitin direct A20 inhibition of inflammation. Nature.

[28] Tokunaga F (2012). Specific recognition of linear polyubiquitin by A20 zinc finger 7 is involved in NF-κB regulation: A20 ZF7 binds linear di-Ub and regulates NF-κB. EMBO J..

[29] Bosanac I (2010). Ubiquitin binding to A20 ZnF4 is required for modulation of NF-κB signaling. Mol. Cell.

[30] Wertz IE (2004). De-ubiquitination and ubiquitin ligase domains of A20 downregulate NF-kappaB signalling. Nature.

[31] Shembade N, Parvatiyar K, Harhaj NS, Harhaj EW (2009). The ubiquitin‐editing enzyme A20 requires RNF11 to downregulate NF‐κB signalling. EMBO J..

[32] Shembade N (2008). The E3 ligase Itch negatively regulates inflammatory signaling pathways by controlling the function of the ubiquitin-editing enzyme A20. Nat. Immunol..

[33] Jin Z (2009). Cullin3-based polyubiquitination and p62-dependent aggregation of caspase-8 mediate extrinsic apoptosis signaling. Cell.

[34] Onizawa M (2015). The ubiquitin-modifying enzyme A20 restricts ubiquitination of the kinase RIPK3 and protects cells from necroptosis. Nat. Immunol..

[35] Yamaguchi N, Yamaguchi N (2015). The seventh zinc finger motif of A20 is required for the suppression of TNF-α-induced apoptosis. FEBS Lett..

[36] De A, Dainichi T, Rathinam CV, Ghosh S (2014). The deubiquitinase activity of A20 is dispensable for NF- B signaling. EMBO Rep..

[37] Polykratis Apostolos, Martens Arne, Eren Remzi Onur, Shirasaki Yoshitaka, Yamagishi Mai, Yamaguchi Yoshifumi, Uemura Sotaro, Miura Masayuki, Holzmann Bernhard, Kollias George, Armaka Marietta, van Loo Geert, Pasparakis Manolis (2019). A20 prevents inflammasome-dependent arthritis by inhibiting macrophage necroptosis through its ZnF7 ubiquitin-binding domain. Nature Cell Biology.

[38] Rodriguez DA (2016). Characterization of RIPK3-mediated phosphorylation of the activation loop of MLKL during necroptosis. Cell Death Differ..

[39] Vereecke L (2010). Enterocyte-specific A20 deficiency sensitizes to tumor necrosis factor–induced toxicity and experimental colitis. J. Exp. Med..

[40] Polykratis A (2014). Cutting edge: RIPK1 Kinase inactive mice are viable and protected from TNF-induced necroptosis in vivo. J. Immunol..

[41] Newton K, Sun X, Dixit VM (2004). Kinase RIP3 is dispensable for normal NF-kappa Bs, signaling by the B-cell and T-cell receptors, tumor necrosis factor receptor 1, and Toll-like receptors 2 and 4. Mol. Cell. Biol..

[42] Verstrepen L (2010). Expression, biological activities and mechanisms of action of A20 (TNFAIP3). Biochem. Pharmacol..

[43] Dondelinger Y (2013). RIPK3 contributes to TNFR1-mediated RIPK1 kinase-dependent apoptosis in conditions of cIAP1/2 depletion or TAK1 kinase inhibition. Cell Death Differ..

[44] Hrdinka Matous, Fiil Berthe Katrine, Zucca Mattia, Leske Derek, Bagola Katrin, Yabal Monica, Elliott Paul R., Damgaard Rune Busk, Komander David, Jost Philipp J., Gyrd-Hansen Mads (2016). CYLD Limits Lys63- and Met1-Linked Ubiquitin at Receptor Complexes to Regulate Innate Immune Signaling. Cell Reports.

[45] Legarda D (2016). CYLD proteolysis protects macrophages from TNF-mediated auto-necroptosis induced by LPS and licensed by type I IFN. Cell Rep..

[46] Sheridan C (2019). Death by inflammation: drug makers chase the master controller. Nat. Biotechnol..

[47] Dondelinger Y (2019). Serine 25 phosphorylation inhibits RIPK1 kinase-dependent cell death in models of infection and inflammation. Nat. Commun..

[48] Sato T, Clevers H (2013). Primary mouse small intestinal epithelial cell cultures. Methods Mol. Biol..

[49] Priem D, Dondelinger Y, Bertrand MJM (2018). Monitoring RIPK1 phosphorylation in the TNFR1 signaling complex. Methods Mol. Biol..

