# Correction: A20 protects cells from TNF-induced apoptosis through linear ubiquitin-dependent and -independent mechanisms

**DOI:** 10.1038/s41419-020-2260-3

**Published:** 2020-01-23

**Authors:** Dario Priem, Michael Devos, Sarah Druw, Arne Martens, Karolina Slowicka, Adrian T. Ting, Manolis Pasparakis, Wim Declercq, Peter Vandenabeele, Geert van Loo, Mathieu J. M. Bertrand

**Affiliations:** 1 Center for Inflammation Research, VIB, Ghent, Belgium; 2 Department of Biomedical Molecular Biology, Ghent University, Ghent, Belgium; 3 Precision Immunology Institute, Icahn School of Medicine at Mount Sinai, New York, NY USA; 4 Institute for Genetics, Cologne Excellence Cluster on Cellular Stress Responses in Aging-Associated Diseases (CECAD) and Center for Molecular Medicine, University of Cologne, Cologne, Germany

**Keywords:** Ubiquitins, Apoptosis, Stress signalling

**Correction to:Cell Death and Disease**

10.1038/s41419-019-1937-y published online 18 September 2019

The original version of this article contained an error in the name of one of the co-authors (Wim Declercq). This has been corrected in the PDF and HTML versions.

