# Supplementary figures and images for: A20 protects cells from TNF-induced apoptosis through linear ubiquitin-dependent and -independent mechanisms
Source: Cell Death Dis. 2019 Sep 18;10(10):692. doi: 10.1038/s41419-019-1937-y (PMC6751190; doi:10.1038/s41419-019-1937-y)

Figure S1

A

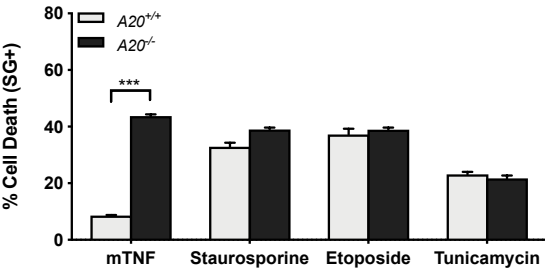

B

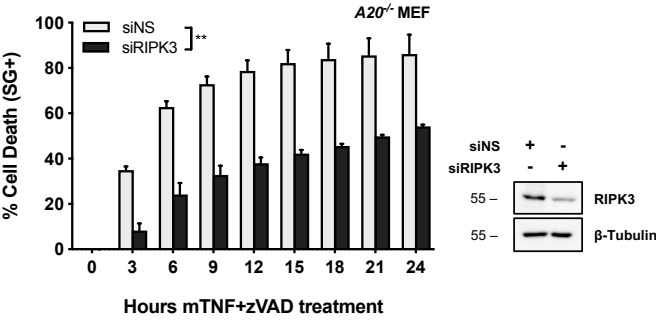

C

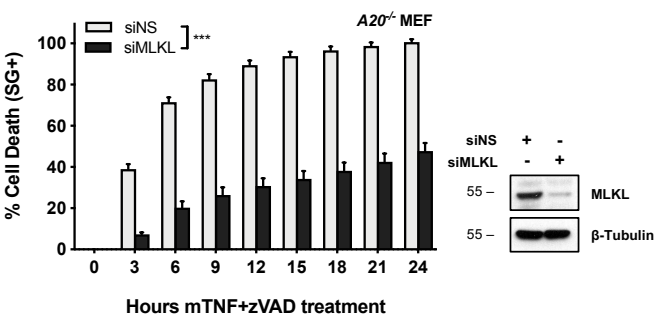

D

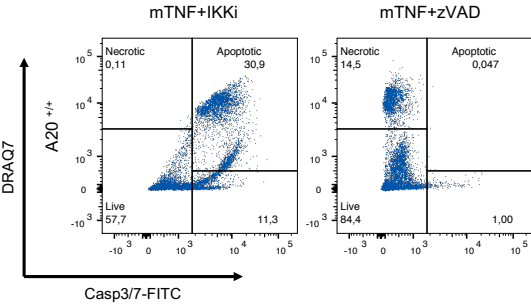

E

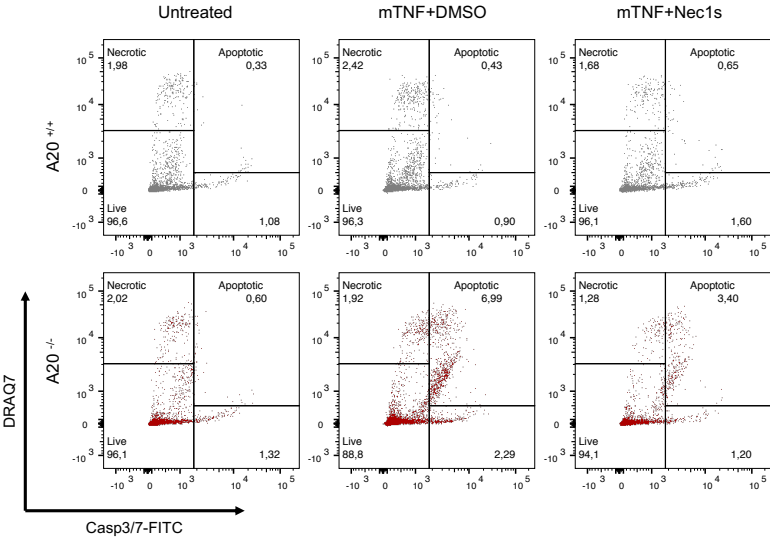

Supplement: Supplementary file 1 — Supplementary Figure 1 [file 41419_2019_1937_MOESM1_ESM.pdf]

Figure S2

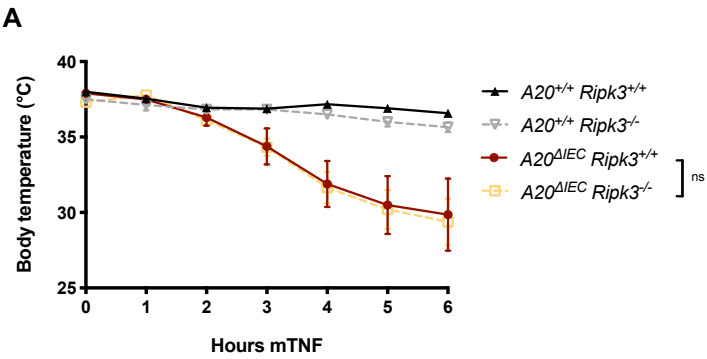

Supplement: Supplementary file 2 — Supplementary Figure 2 [file 41419_2019_1937_MOESM2_ESM.pdf]

Figure S3

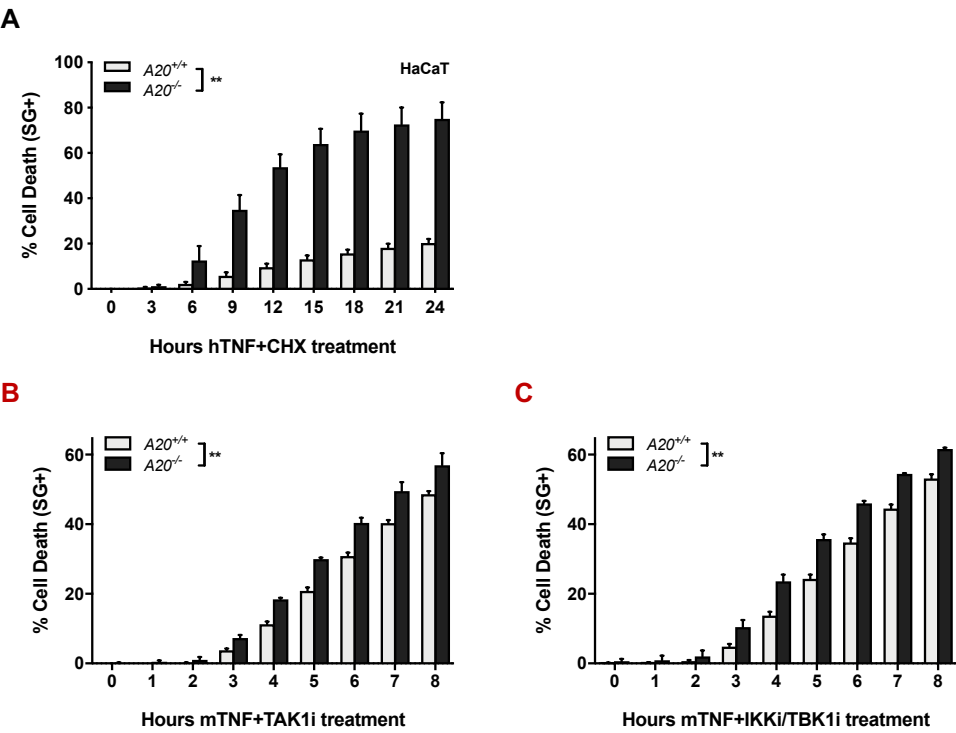

Supplement: Supplementary file 3 — Supplementary Figure 3 [file 41419_2019_1937_MOESM3_ESM.pdf]

Figure S4

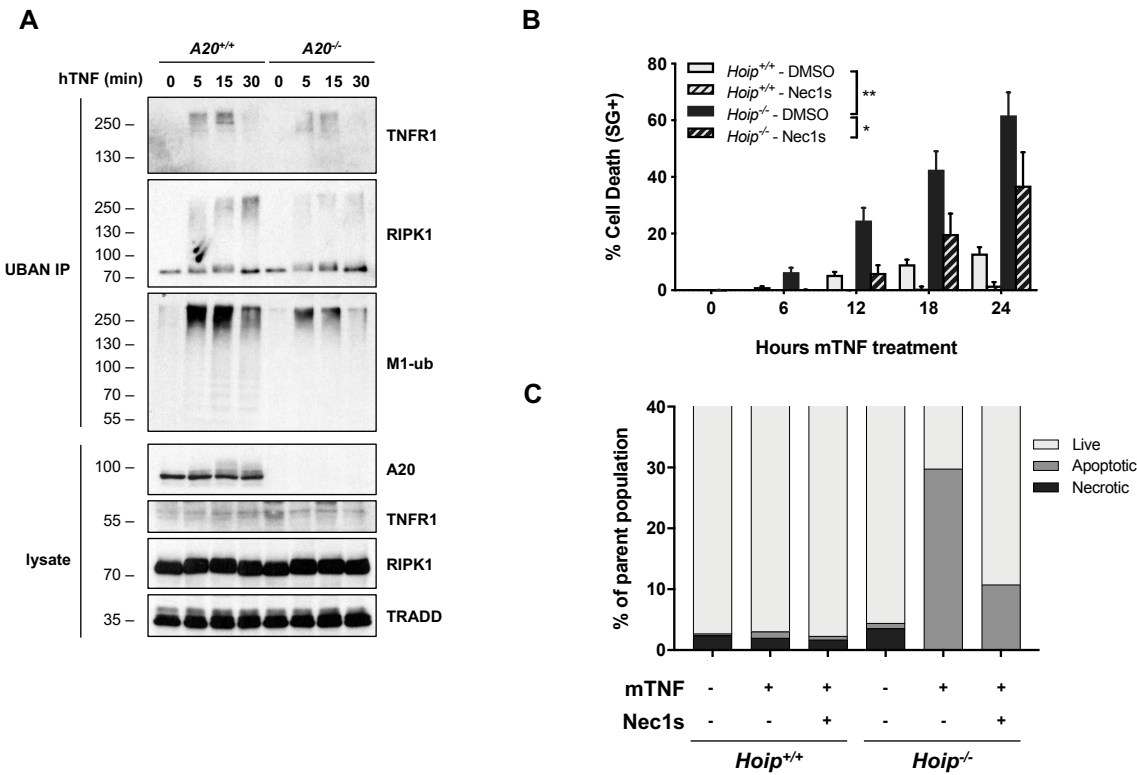

Supplement: Supplementary file 4 — Supplementary Figure 4 [file 41419_2019_1937_MOESM4_ESM.pdf]

Figure S5

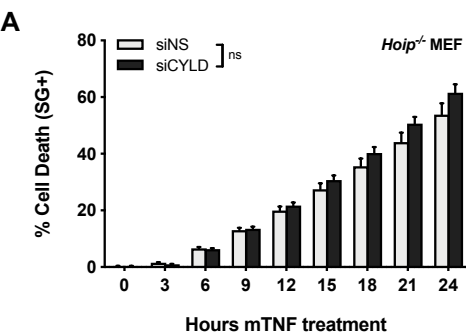

Supplement: Supplementary file 5 — Supplementary Figure 5 [file 41419_2019_1937_MOESM5_ESM.pdf]
